# Endoscopic retrieval of gastric trichobezoar after fragmentation with electrocautery using polypectomy snare and argon plasma coagulation in a pediatric patient

**DOI:** 10.1093/gastro/gov013

**Published:** 2015-04-15

**Authors:** Mohammed Amine Benatta

**Affiliations:** Digestive Endoscopy Unit, Military Central Hospital, Algiers, Algeria

**Keywords:** trichobezoar retrieval, endoscopic fragmentation

## Abstract

Trichobezoars are rare, composed of hair and more common in female pediatric patients with psychiatric disorders. Open surgical extraction is the most common removal method. Only two endoscopic removals after fragmentation have been reported in the English literature. We report herein a third case of a trichobezoar that was successfully retrieved after endoscopic fragmentation with snare polypectomy and argon plasma coagulation in a six-year-old female patient. The trichobezoar mass was completely removed in 10 pieces after 15 passes. The patient’s course was uneventful. Nine months later, she is doing well. This endoscopic removal was effective, minimally invasive and time saving.

## Introduction

The term bezoar refers to swallowed indigestible materials that collect in the gastrointestinal tract, leading to formation of a mass that fails to pass through the intestines. The first published description of bezoars was by Baudamant in 1779 [1]. Most bezoars are commonly found in the stomach. Trichobezoars are rare, composed of hair and more common in female pediatric patients with psychiatric disorders. Bezoars that extend through the pylorus in the form of a tail into the small bowel are known as the Rapunzel syndrome. Trichobezoars may cause potentially life-threatening complications such as intestinal obstruction, gastric bleeding, and perforation [2]. They must be removed once the diagnosis is made, especially when they become embedded in the gastric mucosa. Gastric trichobezoars may attain large sizes before becoming symptomatic. The most common removal method for a large trichobezoar is epigastric surgical incision. The treatment approach depends on the type of bezoar and its location. Of five endoscopic trichobezoar removals with fragmentation, only two have been reported in the English literature [3–7]. We report herein a third case of a trichobezoar that was successfully retrieved after endoscopic fragmentation with snare polypectomy and argon plasma coagulation (APC) in a female patient.

## Case presentation

**Figure 1.**
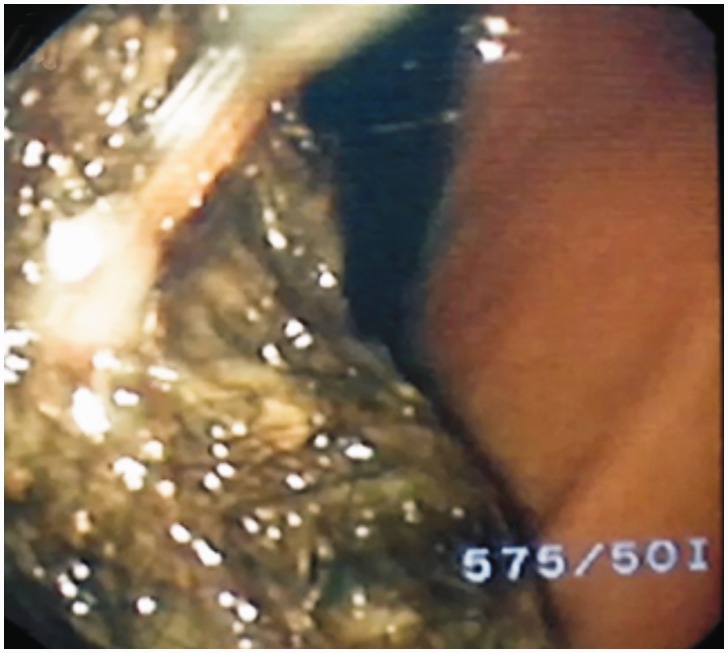
Large intragastric trichobezoar at endoscopy

A six-year-old girl presented with abdominal pain. She had no prior history of medical problems or mental disturbance. Physical examination showed a healthy girl. There was mild tenderness in the epigastric region, although no abdominal mass was palpable. An upper endoscopy showed a gastric trichobezoar. Laboratory data were within normal limits except for anemia. Therefore, she was sent to our hospital for further investigation and surgical treatment. Abdominal ultrasound revealed a gastric foreign body, and barium follow-through revealed a filling defect of the fundus and the gastric body. An endoscopic second look was scheduled with the pediatric surgeon in the operating room to decide on the appropriate management. The endoscopic finding was a large intragastric trichobezoar (Figure 1) about 8 cm × 4 cm in size with extension of a few hairs through the pylorus. We decided to attempt an endoscopic extraction.

**Figure 2.**
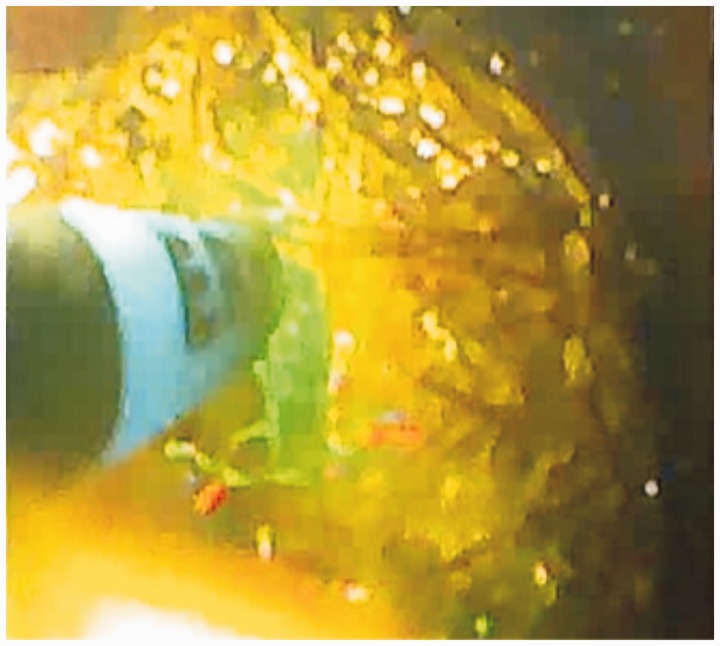
Trichobezoar fragmentation by argon plasma coagulation

**Figure 3.**
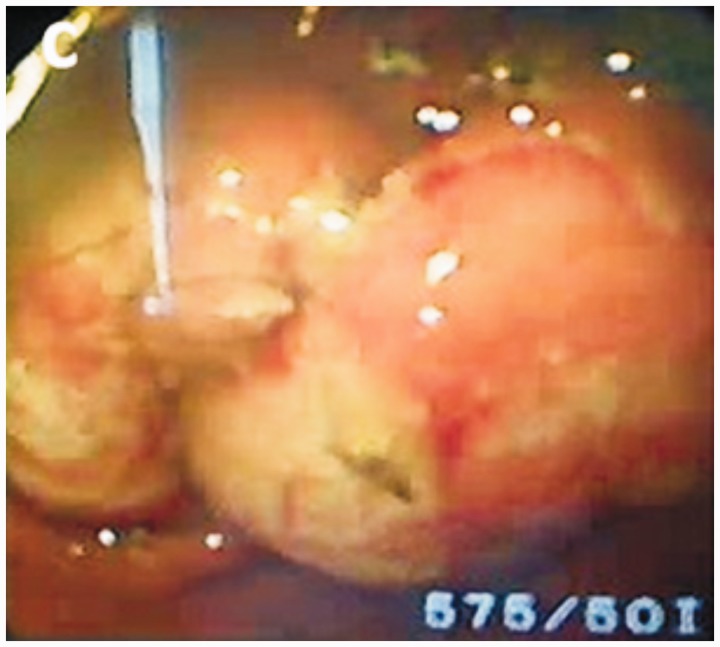
Mucosal polypoid ulceration at the embedding site in the greater curvature

**Figure 4.**
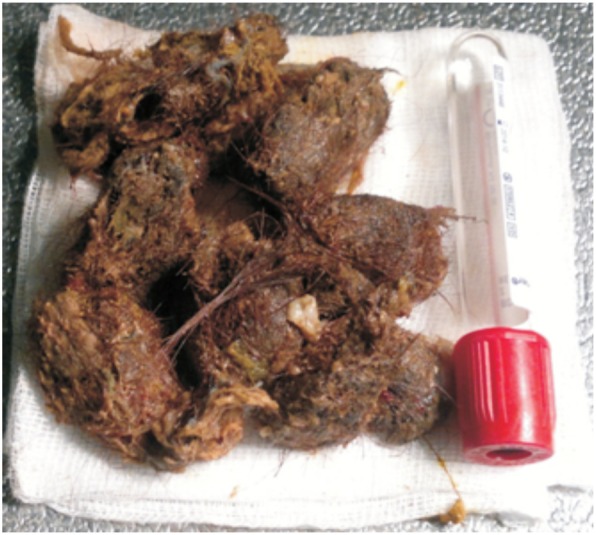
The trichobezoar mass, completely removed after fragmentation into 10 pieces

The endoscopic procedure was conducted under general anesthesia. A standard gastroscope (GIFQ 180; Olympus) was used. Removal using a retrieval basket was not possible. The trichobezoar was large and embedded in the gastric mucosa. Subsequently, the bezoar was fragmented into pieces using alternate polypectomy snare to cut through the trichobezoar and APC (ERBE, VIO, 200 D) (settings: effect 2; 40 W) (Figure 2). Small fundic erosions were noted, with mucosal polypoid ulceration at the embedding site in the greater curvature (Figure 3). The trichobezoar’s tail was pulled back from the duodenal bulb, and a duodenal linear laceration was noted. The total operating time was 50 minutes. The trichobezoar mass was completely removed endoscopically in 15 passes after fragmentation into 10 pieces (Figure 4). The patient’s course was uneventful. She was pain free and tolerated a regular diet by the next morning. Trichotillomania was diagnosed on psychiatric evaluation and treated accordingly. Nine months later, she is doing well.

## Discussion

Many approaches have been proposed for the management of trichobezoars. Endoscopic retrieval or surgical removal is chosen according to the size and composition of the bezoar. Most of the reported cases have required surgery, and a case of trichobezoar treated successfully with gastroscopic technique assisted by laparoscopy was reported [8]. Endoscopic removal is usually not possible when the trichobezoar is embedded into the gastric mucosa, as in our case. To the best of our knowledge, five previous endoscopic attempts to remove trichobezoars have been reported in the English literature [3–7]. The first report was a failure, despite multiple endoscopic sessions with use of an Nd:YAG laser and extracorporeal shock-wave lithotripsy [3]. Subsequently, four cases of endoscopic trichobezoar removal were reported, including two cases after fragmentation [4–7]. Soehendra used a Nd:YAG laser; the retrieval of fragments required more than 100 passages of the endoscope in three sessions of two to three hours each [4]. Aybar used a hot biopsy forceps and a snare (ERBE Apc230) (settings: effect 2–4; 30–40 W). The trichobezoar was 8 cm × 7 cm in size and was fragmented into 13 pieces and completely removed after 25 passes [7]. The procedure time was three hours. In our case, removal of the 10 pieces was obtained by alternately using a combination of polypectomy snare and APC. The procedure was carried out under general anesthesia with no complications, and the total operating time was only 50 minutes. This is the third reported case of a trichobezoar that was successfully retrieved after endoscopic fragmentation. When a bezoar is deemed impossible to remove in one piece, fragmentation should be attempted.

In our case, complete endoscopic removal of an embedded trichobezoar was carried out in less than one hour after fragmentation using electrocautery with a combination of polypectomy snare and APC. This endoscopic removal was effective, minimally invasive, and time saving. We thought that this approach should be considered as an alternative to surgery in selected cases.

*Conflict of interest statement:* none declared.
